# The Association Between Physical Activity, Mental Status, and Social and Family Support with Five Major Non-Communicable Chronic Diseases Among Elderly People: A Cross-Sectional Study of a Rural Population in Southern China

**DOI:** 10.3390/ijerph121013209

**Published:** 2015-10-21

**Authors:** Xiang Huang, Huajie Yang, Harry H.X. Wang, Yongjun Qiu, Xiujuan Lai, Zhiheng Zhou, Fangjian Li, Liwei Zhang, Jiaji Wang, Jimin Lei

**Affiliations:** 1 School of Public Health, Guangzhou Medical University, Guangzhou 510182, China; E-Mails: mynameishx@163.com (X.H.); laixj66@163.com (X.L.); zhihengz@163.com (Z.Z.); lifangjian7502@163.com (F.L.); zlw1980@126.com (L.Z.); 2 The Sanxiang Community Health Service Center of ZhongShan City, Zhongshan 528463, China; E-Mail: mythyhj@163.com; 3 School of Public Health, Sun Yat-Sen University, Guangzhou 510080, China; E-Mail: Haoxiang.Wang@glasgow.ac.uk; 4 General Practice and Primary Care, Institute of Health and Wellbeing, University of Glasgow, Glasgow G12 9LX, UK; 5 The Health and Family Planning Bureau of ZhongShan City, Zhongshan 528403, China; E-Mail: 13702372004@163.com

**Keywords:** non-communicable chronic disease, activities of daily living, mental status, social relationship

## Abstract

*Background*: Non-communicable chronic diseases (NCDs) have become the top threat in China. This study aimed to estimate the prevalence of major NCDs among the elderly population in rural areas in southern China and explore its associated social determinants. *Methods*: A multistage cluster random sampling methodology was adopted to select a total of 9245 rural elderly people from 3860 rural households in Guangdong Province. Interviews and physical examinations were performed to collect patient information. Descriptive and logistic regression analyses were conducted to explore factors associated with the presence of major NCDs. *Results*: Over one-third (38.5%) of the study population suffered from five major NCDs. The grade of activities of daily living (ADL), mental status, and social relationship of elderly people without NCDs were better than those with NCDs. The major factors associated with the presence of NCDs among the elderly people included age (70–79 years group and 80–89 years group), education level (senior high/technical secondary school and junior college and above), mental status (concentration, enrichment and happy life and memory), relationship with neighbours, activities of daily living (ADL) (being able to climb three floors and bend over), physical activity, marital status (bereft), and living conditions (with offspring and family members). *Conclusions*: The study identified several social determinants associated with the presence of major NCDs. A higher level of family support and physical exercise might contribute to improved physical condition, mental status, and ADL among the elderly people in rural areas in southern China.

## 1. Introduction

With the worsening global environment and the increasing prevalence of aging, non-communicable chronic diseases (NCDs) have become the main health problem worldwide [1,2]. In developed countries, NCDs now account for an estimated 80% of total deaths and 70% of the total number of disability-adjusted life-years (DALYs) [3], and it is expected to serve as the major cause of global death and disability by 2020 [4,5]. In China, the occurrence of NCDs is increasing yearly. NCDs such as cerebrovascular diseases, cancer, heart diseases, and chronic respiratory diseases have caused most of the burden of ill health, exceeding the burden caused by traditional communicable diseases. The NCD-related medical costs account for approximately 70% of the total health care expenditures [6,7]. Therefore, studies on the prevention and control of NCDs are urgently needed.

Aging is also a very serious social problem globally. It has been reported that the number of people aged sixty years and above is estimated to increase by more than triple, especially in developed countries, where elderly people are expected to account for at least one-third of the total population within the next fifty years [8]. As shown in many studies, aging leads to a series of problems, with the most concern being the escalating medical expenditures and higher morbidity and mortality [9,10]. It is noted that those problems tend to be worse in rural areas than that in urban areas due to unbalanced economic development and an unequal distribution of health care resources [11,12,13].

During the last two decades, a large number of studies have been conducted to elucidate the factors associated with NCDs. Those studies found that NCDs represented a complex concomitant of biological, social and psychological factors, but the predominant point is that NCDs are mainly associated with lifestyle. For example, studies have demonstrated that people who had stronger social support were less likely to suffer from NCDs, and people with well social support were more likely to develop positive health behaviours, including better adherence to medical treatment regimes, exercise, healthy diet, and smoking cessation [14]. Mental status, including feelings of enrichment and happy life, depression [15], loneliness [16], memory and concentration were also associated with NCDs since it was usually related with the neurophysiological system of human beings, which play a more important role in stabilizing the internal environment.

Previous studies have also shown that physical exercise could be linked to longevity regardless of genetic factors. Even at an older age, physical activity (PA) can significantly reduce the risk of coronary heart disease, diabetes, high blood pressure, and obesity, help to reduce stress, anxiety and depression, and improve lipid profile [17]. PA can also reduce the risks of colon cancer, breast cancer and ischemic stroke [18,19]. A moderate level of PA for more than one hundred and fifty minutes or vigorous PA of sixty minutes per week at gym, home, or elsewhere can reduce the risk of developing coronary heart disease by approximately 30% [17]. However, under the context of industrialization and urbanization, the mechanized transportation have largely reduced daily PA in both developed regions and developing countries, and currently more than 60% of the global populations are considered to have insufficient PA.

With the increasing medical standards and universal health awareness, the concerns for elderly people’s health have become the focus of society. In China, the five major common NCDs including hypertension, diabetes, stroke, heart disease, and chronic obstructive pulmonary disease (COPD) have become an important public health issue that seriously endanger people’s health as the number of deaths from NCDs accounted for more than 80% of deaths due to all illnesses [20]. To reduce the incidence of NCDs, strategies focusing on the drivers of the epidemic and social determinants of NCDs must be developed. The presence of co-morbidities and substance use are common among the elderly population, yet the social factors that determine the course of the NCDs epidemic and measurements to quantify exposure to these risk factors have been inadequately researched in China. In this study, we aimed to assess how social factors affected the elderly people suffer from the major NCDs in southern China, thereafter providing evidence-based recommendations for better NCDs management and enriching the international experiences of NCDs control.

## 2. Methods

### 2.1. Study Design and Sample Subjects

We conducted a cross-sectional study to randomly select 9245 rural elderly people (aged ≥60 years) from 3860 rural households in Guangdong Province in southern China. The baseline information of the recruited subjects was collected from March to September in 2010 using a valid questionnaire. The study was approved by the Ethics Review Committees of Guangzhou Medical University in China. All participants provided voluntary informed consent.

After China’s new round of medical and health care system reform in 2009, the Chinese government has turned to build a community health service (CHS) network that will allow residents to access primary care within a fifteen-minute walking distance and to provide more reasonable medical coverage, and every CHS facility is responsible for the service of several communities [21,22]. According to the social and economic development level, Guangdong Province is divided into four regions: northern area, central area (the Pearl River Delta), eastern area, and western area. A multi-stage cluster random sampling method was used to recruit the elderly resident population from the agency which were selected randomly from different geographic regions in Guangdong Province. In the first stage, nine cities were selected, including two cities in the west, two cities in the north, two cities in the east and three cities in the central. In the second stage, one CHS facility was chosen randomly according to their registered code in each city. In the third stage, five villages were selected within the service area for each agency. Accordingly, forty-five villages were randomly selected from each of the nine randomly selected agencies. The total sample size in the study was 9245. The inclusion criteria of the study subjects were: (1) Subjects aged sixty years and above; (2) Regular residents who have lived in these committees for at least six months. 

Both qualitative (observation and interview) and quantitative (questionnaire survey) methods were used. The questionnaires collected self-reported information on the subjects’ basic demographic data and their current community social support. The household face-to-face interviews were conducted by well-trained interviewers who were composed of undergraduate students at Guangzhou Medical University and community medical staff trained by the principal investigator.

### 2.2. Measurement Tools

The community doctors and nurses conducted the physical examinations. We documented all self-reported chronic diseases that were either diagnosed previously at any hospital or newly identified on-site during the study. This study focused on five major NCDs, including hypertension, diabetes, stroke, heart disease, and chronic obstructive pulmonary disease (COPD), which are prevailing in the community threatening people’s health [23]. Subjects with chronic health status were defined as those who were diagnosed with at least one of the chronic disease above at hospitals or who were found having a chronic disease during the survey. 

The chronic diseases were recorded according to the disease classification standard of outpatient service in the government annual report established by the Ministry of Health, P.R. China. The diagnosis standard of essential hypertension was in accordance with the 1999 World Health Organization International Society of Hypertension (WHO-ISH) guidelines for hypertension management. Hypertension was defined as systolic blood pressure (SBP) ≥140 mmHg and/or diastolic blood pressure (DBP) ≥90 mmHg (or SBP ≥130 mmHg/DBP ≥80 mmHg for those with concomitant diabetes). Patients with diabetes, COPD, stroke and heart disease were determined according to previous hospital diagnostics.

The questionnaire development [24] included literature review, focus group discussions, construction of items, pilot study, followed by a formal study to investigate preliminary validity and reliability. Focus group discussions were held among twelve specialists and eight local community medical staff. The professional areas of the specialists covered chronic disease management, epidemiology, CHS, and psychology. Focus group discussions were led by a trained moderator who adhered to a pre-defined question guide. The first theme of questions asked about the PA of the participants, and the second theme asked about the three aspects about family support including activities of daily living (ADL), mental status, and social relationship. The discussions were recorded, transcribed verbatim, and processed as text until no new themes emerged. A qualitative content analysis was conducted.

The item pool for the questionnaire was generated from the themes associated with major NCDs among elderly population on the PA, mental status, and social relationships that were discussed in the focus groups. In addition, we conducted a literature review and solicited expert opinions (provided by a panel comprising three experts of NCDs management, two psychologists, three CHS experts, three epidemiologists, two physicians and two nurses). After the items were written, the focus groups were used again to discuss whether the items were relevant, clear, unambiguous, and written in language that would be understandable to potential respondents. Items with an endorsement rate lower than 0.2 were discarded. The face validity was assessed by five specialists in nursing, public health, and statistics. The content validity index (CVI) of the questionnaire was 0.92. Reliability of the questionnaire was assessed by one hundred subjects in Zhongshan city of Guangdong Province. The Cronbach’s alpha score was 0.88 and the intra-class correlation coefficient (ICC) was 0.90, showing good international reliability. The finial questionnaire included demographic information, the PA of the participants, and the three aspects about family support. 

We used a questionnaire regarding family relationships which asked the following 11 questions. ADL questions included: (1) Can you do the housework last more than one hour? (2) Can you climb three floors only by yourself? (3) Is there any problem you bend over, knee bending or squat? (4) Can you walk one km yourself? Mental status questions included: (5) Do you feel your life enrichment and happy? (6) Do you always feel depression or dejected? (7) How about your memory? (8) Can you concentrate on one thing at a time for more than ten minutes? Social Relationship questions included: (9) How about you family relationship? (10) How about your relationship with relatives? (11) How about your relationship with neighbours? 

For the ADL questions, we asked each respondent to rate the level with five-point scales (0 = “starkly unable”, 1 = “probably unable”, 2 = “half unable”, 3 = “a little problem”, 4 = “no problem”), mental status with five-point scales (0 = “none at all”, 1 = “occasionally have”, 2 = “sporadic”, 3 = “regularly have”, 4 = “always have”), and the social relationship with a six-point scale (5 = “excellent”, 4 = “good”, 3 = “average”, 2 = “rather poor”, 1 = “poor”, 0 = “none communication”). For the reversed scales of some items, we still adopted the scales on the basis of whether it is the positive items or not, especially for the mental status. The higher scores in elderly people indicated the self-reported better health conditions.

Points were given based on the answers to these questions. For the questions about ADL and mental status, subjects were classified into three grades according to the scores in order to indicate the level of situations: low (0–7), moderate (8–11), and high (12–16). For the social relationship, subjects were classified into two grades according to the scores in order to indicate the level of situations: suboptimal (0–11) and good (12–15).

Health status was assessed on the basis of response to a self-perceived statement “How do you evaluate your present health condition?’’ (1 = “excellent”, 2 = “good”, 3 = “average”, 4 = “rather poor”, 5 = “poor”). In this study, subjects who responded “excellent” or “good” were considered as having good self-perceived health status, and those who responded else were considered as having a suboptimal self-perceived health status.

PA has been defined as “any bodily movement produced by skeletal muscles that resulted in energy expenditure”. We divided PA into aerobic and anaerobic activity by the following criterion: twenty–sixty minutes of moderate- to high-intensity endurance exercise (60%–90% of the maximum heart rate or 50%–85% of the maximal aerobic power) performed three or more times per week. We counted Tai-Chi, gymnastics, walking, hiking, dancing, playing basketball, swimming, bicycling, yoga, and jogging, all of which are common aerobic PA in China. 

### 2.3. Statistical Analysis

All questionnaires were checked for completeness. The EpiData software (version 3.1) was used for data entry. Double entry was conducted to ensure data accuracy. The SAS system (version 8.2; SAS institute Inc., Cary, NC, USA) was used for data cleaning and analysis. The data were presented as rates, and differences between groups examined using the chi-square test. All alpha levels were set to 0.05. The logistic regression analysis was performed to analyze factors associated with the presence of five major NCDs among the rural elderly people. Variable selection for multiple regression analyses was conducted using a stepwise entry process, and the presence of NCDs was input as the dependent variable (1 = yes, 0 = no). A univariate logistic regression analysis was conducted, considering fourteen related factors including age, gender, marital status, education, smoking habit, alcohol consumption, PA, employment status, economic status, living arrangement, subjective health, ADL, mental status and social relationship. Factors demonstrated to be statistically significant in the univariate analysis were entered into a multivariate logistic regression as independent variables in order to analyze the influencing factors associated with the presence of five major NCDs among the rural elderly population, and the backward LR method was used to build the regression model with 0.05 as the inclusion standard and 0.10 as the exclusion standard.

### 2.4. Ethical Approval

All research was conducted with integrity and in line with generally accepted ethical principles and approved by Research Ethic Committee of Guangzhou Medical University in China. All participants read a statement that explained the purpose of the survey and gave written informed content before participation in the study. 

## 3. Results

### 3.1. Demographic Characteristics of Diseased and Non-diseased Elderly People

Of these 9245 rural elderly people aged sixty years and above, 3559 (38.5%) people were suffering from major NCDs included in the study. We divided the participants according to the presence of NCDs. The average age of the elderly people in the diseased and non-diseased group was 72.30 and 69.98 years, respectively. The demographic information between the two groups differed significantly in terms of age (*p* < 0.001), smoking habit (*p* < 0.001), alcohol drinking (*p* < 0.001), PA (*p* < 0.001), marital status (*p* < 0.001), education (*p* < 0.001), employment status (*p* < 0.001), economic status (*p* < 0.001), living arrangement (*p* < 0.001) and self-rated health status (*p* < 0.001) (Table 1).

**Table 1:** Comparison of demographic characteristics between diseased group and none-diseased group of the elderly people, n (n = number, %).

Parameters	Diseased Group (n = 3559)	None-Diseased Group (n = 5686)	χ²	*p*
**Gender**				
Male	1659 (46.6)	2729 (48.0)	1.67	0.196
Female	1900 (53.4)	2957 (52.0)		
**Age (years)**				
60–69	1482 (41.6)	3173 (55.8)	185.52	<0.001
70–79	1288 (36.2)	1665 (29.3)		
80–89	677 (19.0)	738 (13.0)		
≥90	112 (3.1)	110 (1.9)		
Mean ± SD	72.30 ± 8.59	69.98 ± 9.55		
**Smoking habit**				
Never smoked	2396 (67.4)	4022 (70.8)	141.71	<0.001
Current smoker	827 (23.2)	1464 (25.7)		
Ex-smoker	336 (9.4)	200 (3.5)		
Smoking period of current smokers(Mean ± SD)(years)	42.11 ± 13.37	38.83 ± 13.64		
**Alcohol drinking**				
Seldom	2877 (80.9)	4375 (76.9)	26.21	<0.001
Sometimes, usually, always	682 (19.1)	1311 (23.1)		
Drinking period(Mean ± SD)(years)	36.68 ± 15.25	33.29 ± 14.27		
**Physical activity (PA)**				
None	2150 (60.4)	3765 (66.2)	44.74	<0.001
Anaerobic PA	127 (3.6)	248 (4.4)		
Aerobic PA	1282 (36.0)	1673 (29.4)		
**Marital status**				
Unmarried	63 (1.8)	107 (1.9)	70.56	<0.001
Married	2296 (64.5)	4106 (72.2)		
Divorced	13 (0.4)	33 (0.6)		
Bereft	1187 (33.3)	1440 (25.3)		
**Education**				
Illiteracy and Primary school	3014 (84.7)	4726 (83.1)	22.20	<0.001
Junior high school	385 (10.8)	743 (13.1)		
Senior high/Technical Secondary school	122 (3.4)	172 (3.0)		
Junior college and above	38 (1.0)	45 (0.8)		
**Employment status**				
Employed	769 (21.6)	1642 (28.9)	60.02	<0.001
Unemployed, retired	2790 (78.4)	4044 (71.1)		
**Economically**				
Dependent	1279 (35.9)	2169 (38.1)	1619.66	<0.001
Independent	2280 (64.1)	3517 (61.9)		
**Living arrangement**				
Only with spouse	573 (16.1)	803 (14.1)	52.92	<0.001
Only with offspring	1125 (31.6)	1695 (29.8)		
With family members	1416 (39.8)	2656 (46.7)		
Living alone	381 (10.7)	447 (7.9)		
Others	64 (1.8)	85 (1.5)		
**Subjective health**				
Good	767 (21.6)	3085 (54.3)	2719.16	<0.001
Suboptimal	2792 (78.4)	2601 (45.7)		

The results showed that the prevalence of hypertension was the highest among the five major NCDs in the different age groups and genders, and the prevalence of COPD was the lowest. In general, the prevalence of hypertension, COPD, stroke and heart disease increased with ageing, although the opposite trend was observed for diabetes. The prevalence of five major NCDs among rural elderly people differed significantly by gender and age (*p* < 0.001) (Table 2).

**Table 2:** The prevalence of major NCDs among rural elderly people by age, gender [*n*(%)].

Items	Total	Hypertension	Diabetes	COPD	Stroke	Heart Disease	χ²	*p*
**Gender**								
Male	1659 (100.0)	1165 (70.3)	163 (9.8)	95 (5.7)	114 (6.9)	128 (7.7)	50.931	<0.001
Female	1900 (100.0)	1329 (69.9)	216 (11.4)	49 (2.6)	57 (3.0)	170 (8.9)		
**Age (years)**								
60–69	1482 (100.0)	1012 (68.3)	196 (13.2)	39 (2.6)	68 (4.6)	102 (6.9)	77.165	<0.001
70–79	1288 (100.0)	917 (71.2)	144 (11.2)	54 (4.2)	64 (5.0)	118 (9.2)		
80–89	677 (100.0)	496 (73.3)	33 (4.9)	40 (5.9)	35 (5.2)	56 (8.3)		
≥90	112 (100.0)	72 (64.3)	7 (6.2)	11 (9.8)	4 (3.6)	22 (19.6)		
**Total**	3559 (100.0)	2494 (70.1)	379 (10.6)	185 (4.0)	202 (4.8)	299 (8.4)		

In the different age groups, rural elderly people aged above 60 years showed a higher rate of developing major NCDs. The proportion of people who suffered from three or more major NCDs in the age group (60–69 years) was 45.5% (for males) and 14.9% (for females), respectively (**χ^2^** = 2933.719, *p* < 0.001). The proportion of those with three or more major NCDs among subjects aged between 70–79 years was 39.0% (for males) and 59.6% (for females) (**χ^2^** = 5.898, *p* = 0.117). We observed gender-specific differences in the prevalence of NCDs among subjects aged between 60–69 years and 80–89 years (Table 3).

**Table 3:** Number of chronic conditions by age and gender (*n* = number).

Age (Years)	Gender	0	1	2	≥3	χ^2^	*p*
60–69	Male	30 (2.5)	1128 (62.1)	298 (61.6)	56 (45.5)	2933.719	<0.001
	Female	1590 (54.2)	5 (0.6)	6 (2.8)	7 (14.9)		
70–79	Male	819 (68.3)	475 (26.2)	118 (24.4)	48 (39.0)	5.898	0.117
	Female	846 (28.8)	497 (55.9)	122 (56.2)	28 (59.6)		
80–89	Male	321 (26.8)	192 (10.6)	63 (13.0)	13 (10.6)	10.156	0.017
	Female	417 (14.2)	328 (36.9)	71 (32.7)	9 (19.1)		
≥90	Male	30 (2.5)	21 (1.1)	5 (1.0)	6 (4.9)	1.318	0.251
	Female	80 (2.7)	59 (6.6)	18 (8.3)	3 (6.4)		

### 3.2. Family Support Scores

The scores of non diseased elderly people were much better than that of diseased people in ADL (none-diseased group, 71.3% *versus* diseased group 55.0%) and mental status (none-diseased group, 44.2% *versus* diseased group 32.4%) in the grade of high scores, and the proportion of non diseased group (81.5%) among elderly people who have good social relationship was significantly higher than that of diseased group (77.2%). And there was significantly different between the two groups in all of the ADL (**χ^2^** = 338.67, *p* < 0.001), mental status (**χ^2^** = 183.08, *p* < 0.001) and social relationship (**χ^2^** = 25.63, *p* < 0.001) parameters (Table 4).

**Table 4:** Scores of ADL, mental status and social relationship among the rural elderly people, (*n* = number, %).

Parameters	Diseased Group (n = 3559)	None-Diseased Group (n = 5686)	χ²	*p*
**ADL**				
low (0–7)	824 (23.2)	586 (10.3)	338.67	<0.001
moderate (8–11)	776 (21.8)	1046 (18.4)		
high (12–16)	1959 (55.0)	4054 (71.3)		
Mean ± SD	10.83 ± 4.75	12.62 ± 3.82	—	—
**Mental status**				
low (0–7)	581 (16.3)	514 (9.0)	183.08	<0.001
moderate (8-11)	1824 (51.3)	2661 (46.8)		
high (12–16)	1154 (32.4)	2511 (44.2)		
Mean ± SD	10.08 ± 2.83	10.91 ± 2.46	—	—
**Social Relationship**				
suboptimal (0–11)	813 (22.8)	1052 (18.5)	25.63	<0.001
good (12–15)	2746 (77.2)	4634 (81.5)		
Mean ± SD	12.18 ± 1.98	12.35 ± 1.82	—	—

### 3.3. Determinants of Suffering from Major NCDs Associate with Family Support

The results of the multivariate logistic regression showed that the major influencing factors associated with the presence of five major NCDs among the rural elderly people included: age: 70–79 years group and 80–89 years group; education level: senior high/technical secondary school and junior college and above; mental status: concentration, enriched and happy life and memory; social relationship: relationship with neighbours; ADL: being able to climb three floors, bend over and knee bending, or squat; PA; marital status: bereft; living arrangement: living only with offspring, living with family members. The opportunities of suffering from NCDs among elderly people was increasing with age and education level, and the status of concentration, and the relationship with neighbours and bend over, knee bending or squat could also increase the opportunities of suffering from NCDs. The elderly people who have enrichment and happy life, good memory and ADL, and could climb 3 floors may have the fewer opportunities suffering from NCDs (Table 5).

**Table 5:** Multivariate logistic regression on determinants of suffering from major NCDs.

Parameters	OR	95.0% CI	*p* Value
**Age groups**			
70–79/60–69	1.329	1.198–1.474	0.000
80–89/60–69	1.284	1.113–1.483	0.001
**Education**			
Senior high/technical secondary school/Illiteracy and Primary school	1.409	1.097–1.809	0.007
Junior college and above/Illiteracy and Primary school	2.470	1.841–6.171	0.002
**Mental status**			
Concentration	1.207	1.082–1.347	0.001
Enrichment and happy life	0.761	0.688–0.841	0.000
Memory	0.707	0.638–0.782	0.000
**Social relationship**			
Relationship with neighbours	1.300	1.040–1.624	0.000
**ADL**	0.675	0.599–0.762	0.000
Being able to climb 3 floors	0.820	0.691–0.974	0.024
Bend over, knee bending or squat	1.200	1.021–1.411	0.027
**PA**	0.743	0.686–0.804	0.000
**Marital status**			
Bereft / Unmarried	1.442	1.006–2.068	0.046
**Living arrangement**			
Only with offspring/Only with spouse	0.707	0.599–0.835	0.000
With family members/Only with spouse	0.854	0.750–0.973	0.018

Notes: -2 Log likelihood: 11607.678; Cox & Snell R Square: 0.059; Nagelkerke R Square: 0.080.

## 4. Discussion

With the samples drawn in the study, we estimated that more than one-third (38.5%) of the rural elderly people aged sixty years and above in southern China were suffering from any of the five major NCDs. Of the diseased group, the number of people aged seventy years and above was obviously higher than that of the disease-free group. Some studies have reported that chronic diseases affected people of all ages, and the prevalence of chronic disease was strongly related to age [25,26]. In this study, the result of regression analysis showed similar findings that aging was positively associated with the presence of major NCDs, especially for the 70–79 and 80–89 years age groups. A higher level of education was also associated with the likelihood of developing major NCDs, which might be due to the low proportion of high education among the elderly participants. 

This study also showed that the conditions of ADL, mental status and social relationship among elderly people of the non-diseased group were significantly better than those of the diseased group. The regression analysis showed a positive association between the level of self-perceived life enrichment and happiness and the absence of major NCDs. In addition, those who had a better memory were less likely to suffer from major NCDs. The increase in the psychological concentration was positively correlated with the increased likelihood of developing NCDs, and those who had a better relationship with their neighbours were more likely to suffer from major NCDs. Similarly, some studies on negative social exchange suggested that the presence of social relationships may actually add stress to a person’s life rather than reduce it, especially if the relationship is too demanding, insensitive and interfering, or if those with whom one is in contact suffer from serious problems of their own [27,28].

International studies have indicated that PA can significantly reduce the risk of NCDs [17,29]. In this study, elderly people who engaged in PA or those who could climb three floors by themselves alone were significantly less likely to develop major NCDs, whereas elderly people who could not bend over, bend at the knee, or squat easily were much more likely to suffer from major NCDs, perhaps because these activities were more strenuous for most of the elderly people. 

Many reviews have indicated that spouses were the most studied dyad in the context of health and mortality [30,31]. In particular, a large number of studies have focused on the marital status of divorce, widowhood and spousal hospitalization [30]. These studies indicated that marital status, especially divorce and widowhood was closely related to the health of the elderly people, and was the significant risk factor of their mortality [25,32]. In addition, elderly people who were in a marriage were more likely to exercise if their spouse did so since they may have more chances to perform aerobic PA [33]. Elderly people with a stable marriage may have much more support from their families.

Our study found that the proportion of bereft and people who only lived with a spouse or alone in the diseased group was significantly higher than that of the non-diseased group. The regression analysis results showed that the elderly people who were widowed were more likely to suffer from major NCDs compared to those who were unmarried; compared to elderly people who were living only with their spouse, those living with their offspring and other family members were less likely to suffer from major NCDs.

## 5. Limitations

This study has several limitations. First, we could not establish the causal relationships between family support, PA, and suffering from major NCDs using a cross-sectional study design. Further interventional studies based on the findings in this study might help provide more evidence for the establishment of the relationship, such as the objective health measures of biomarkers [34] to reduce the bias of the patients’ self-reports. Also, information bias might exist since the data collected from the elderly people were self-reported, although on-site physical examinations were conducted to substantiate the presence of chronic diseases. Furthermore, there were other potential factors that were not taken into account in this study. For example, district-level variations existed among the study cities as well as the provider-level disparities in the quality of care provided by different community health centres, as the organizational model of primary care itself may impact primary care quality [35] and may be associated with the health status of the study population.

## 6. Conclusions

This study demonstrated that PA and family support have a positive influence on health status in elderly people. In the field of NCD management and control, the importance of good social and family support on the health status of the elderly rural population should be considered. In the process of the development of CHS in China, person-centred concept of service should be more emphasized in order to resolve the physical, psychological and social problems related to health. We recommend that this should be incorporated into the training plan for the medical staff as a core concept during their daily clinical practice.
